# Supplementary material for: Absolute Quantification of Hepatitis B Core Antigen (HBcAg) Virus-like Particles and Bound Nucleic Acids
Source: Viruses. 2023 Dec 21;16(1):13. doi: 10.3390/v16010013 (PMC10820971; doi:10.3390/v16010013)
Supplement: Supplementary file 1 [file viruses-16-00013-s001.zip › viruses-2775405-supplementary.pdf]

Supplementary Materials

Figure S1: Components of flow-through fraction of silica-SC based extraction of the EasyPure Kit on NAGE. In lane 1 proteinase K, lane 2 binding buffer 5, lane 3 Tris buffer and lane 4 ethanol. NAGE: native agarose gel electrophoresis; SC: spin column

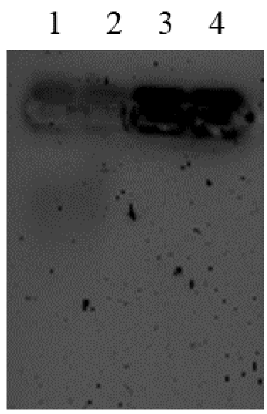

Figure S2: Remaining protein impurities in the extracts after silica-SC based extraction for de novo extractions of CC purified Cp164 VLP with the lysis time of 60 min (lane 3 and 4) on SDS-PAGE for diluted Cp164. Marker in lane 1 and initial Cp164 VLP sample before silica-based extraction in lane 2. CC: CaptoCore 400; SC: spin column; VLP: virus-like particle

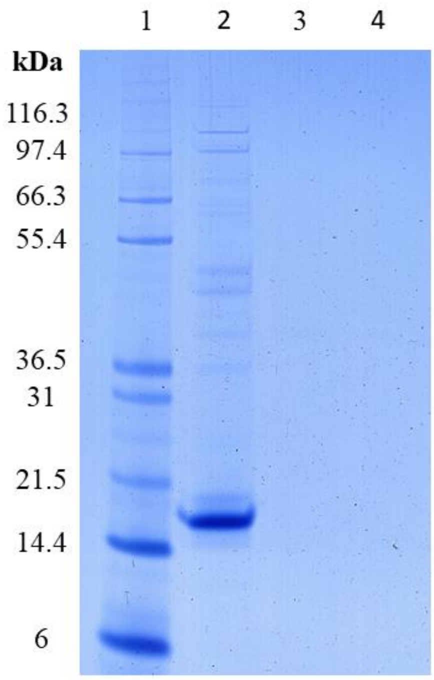

Table S1: Recoveries for preliminary experiments with varying silica-SC based extraction elution procedures, different loads, using Cp157, Cp164 HBcAg VLP constructs and dsDNA, and PicoGreen, RiboGreen assay and qPCR. HBcAg: Hepatitis B core Antigen; VLP: virus-like particle

Elution procedure	Construct / NA	Quantification by	Load [µg]	Recovery [%]
2 x 25 µL	Cp164	RiboGreen	0.275	11.89
4 x 25 µL	Cp164	RiboGreen	0.275	31.74
8 x 25 µL	Cp164	RiboGreen	0.275	53.18
4 x 25 µL	Cp157	PicoGreen	1.929	72.61
4 x 25 µL	Cp157	PicoGreen	1.929	73.18
4 x 25 µL	Cp157	PicoGreen	1.929	72.46
4 x 25 µL	dsDNA	RiboGreen / qPCR	1.139 (RiboGreen) / 0.837 (qPCR)	61.67 / 60.28 (qPCR)
4 x 25 µL	dsDNA	RiboGreen / qPCR	1.139 (RiboGreen) / 0.837 (qPCR)	67.61 / 55.22 (qPCR)
4 x 25 µL	dsDNA	RiboGreen / qPCR	1.139 (RiboGreen) / 0.837 (qPCR)	67.17 / 70.36 (qPCR)
4 x 25 µL	Cp157	RiboGreen	0.814	70.99
8 x 25 µL	Cp157	RiboGreen	0.814	71.03
1 x 200 µL	Cp157	RiboGreen	0.814	77.78
1 x 200 µL	Cp157	RiboGreen	4.67	92.69
1 x 200 µL	Cp157	RiboGreen	2.80	85.21
1 x 200 µL	Cp157	RiboGreen	1.40	81.04
1 x 200 µL	Cp157	RiboGreen	0.47	54.68
1 x 200 µL	dsDNA	qPCR	3.12	85.64

1 x 200 µL	dsDNA	qPCR	1.87	85.29
1 x 200 µL	dsDNA	qPCR	0.94	20.59
1 x 200 µL	dsDNA	qPCR	0.31	64.43

Table S2: Nucleic acid concentrations for Cp157 without and with preliminary silica-SC based extraction and respective standard and relative standard deviation for performed PicoGreen and RiboGreen assay.

**Quantification sample details**	**PicoGreen / RiboGreen**		
	**Concentration in ng/µL**	**Mass in µg**	**relative standard deviation [-]**
VLP without extraction	2.18 ± 0.93 / 5.52 ± 2.55	0.44 / 1.10	42.64 / 46.13
VLP with extraction	1.38 ± 0.13 / 9.65 ± 0.24	0.14 / 0.96	9.08 / 2.51
Recovery extraction #1	1.54 ± 0.22 / 14.01 ± 2.73	0.15 / 1.40	14.43 / 19.49
Recovery extraction #2	1.63 ± 0.14 / 14.12 ± 0.26	0.16 / 1.41	8.58 / 1.87
Recovery extraction #2	1.45 ± 0.24 / 13.98 ± 0.98	0.15 / 1.40	16.78 / 7.01

Table S3: HPLC columns and mobile phases B (A: water + TFA for every condition) of screened in preliminary experiments for the RP based extraction and quantification of HBcAg VLP proteins and bound nucleic acids. HPLC: high performance liquid chromatography; TFA: trifluoroacetic acid; VLP: virus-like particle

HPLC column	Mobile phase B (+ TFA)	% B in binding condition	Outcome
TSKgel Protein C4-300 (3 µm, 4.6x150 mm) from Tosoh Bioscience	acetonitrile	5, 8, 10, 20, 40, 50	High resolution of proteins
	50% acetonitrile / 50% isopropanol	20	
	25% acetonitrile / 75% isopropanol	5, 10, 20, 40, 50	
	isopropanol	20	
BioSuite pPhenyl (10 µm, 4.6x75 mm) from Waters	acetonitrile	5, 10, 20, 40, 50	Low recovery
TSKgel TMS-250 (10 µm, 4.6x75 mm) from Tosoh Bioscience	acetonitrile	5	Low resolution, low recovery
